# Bilateral Bi-Level Erector Spinae Plane Blocks as a Part of Opioid-Sparing Multimodal Analgesia in Scoliosis Surgery: A Case Series of Six Pediatric Patients

**DOI:** 10.3390/medicina59081429

**Published:** 2023-08-07

**Authors:** Malgorzata Domagalska, Bahadir Ciftci, Jerzy Kolasinski, Grzegorz Kowalski, Katarzyna Wieczorowska-Tobis

**Affiliations:** 1 Department of Palliative Medicine, University of Medical Sciences, 61-245 Poznań, Poland; gkowalski@ump.edu.pl (G.K.); kwt@tobis.pl (K.W.-T.); 2 Department of Anesthesiology and Reanimation, Istanbul Medipol University, Istanbul 34214, Turkey; bciftci@medipol.edu.tr; 3 Kolasinski Clinic, Hair Clinic Poznan, 62-020 Swarzędz, Poland; colas@klinikakolasinski.pl

**Keywords:** erector spinae plane block, postoperative pain, multimodal analgesia, scoliosis surgery, pain management

## Abstract

*Background and Aim*: Postoperative pain after scoliosis surgery is severe and usually requires long-term intravenous opioid therapy. Local anesthetic options, such as wound infiltration, are limited and include neuraxial analgesia. However, they are rarely used due to side effects and inconsistent efficacy. We report an opioid-sparing multimodal analgesia regimen with bilateral erector spinae plane blocks. This case series evaluated the analgesic effect of the bilateral bi-level erector spinae plane blocks (ESP) in congenital and neurogenic scoliosis surgery. *Patients and Methods*: Six pediatric patients with congenital or neurogenic scoliosis underwent posterior spinal fusion involving 5 to 12 vertebral levels. Bilateral single-injection ESPB was performed at one or two levels before incision. Preoperatively, patients received intravenous dexamethasone. General anesthesia with endotracheal intubation and volume-controlled ventilation was performed via TIVA with remifentanil and propofol. During and after the procedure, the basic hemodynamic parameters, opioid consumption, pain scores (numerical rating scale/NRS), and possible block complications were monitored. *Results*: All the patients experienced minimal postoperative pain levels. In addition, on the first day after surgery, they had low opioid requirements with no side effects. *Conclusions*: ESPB in patients undergoing congenital and neurogenic scoliosis correction surgery seems to be an essential analgesic technique that may reduce both severities of pain and opioid consumption.

## 1. Introduction

Posterior spinal fusion for scoliosis correction is an excruciating surgery and usually requires long-term, high-dose opioid use for adequate perioperative analgesia [1]. Neuromonitoring, i.e., motor-evoked and somatosensory-evoked potentials (SSEPs), is the current gold standard for preventing neurological damage [2]. Local anesthesia is essential to multimodal analgesia, but options are limited. Intrathecal or epidural opioid injections of local anesthetics have been reported. Still, they are rarely used due to logistical complexity, side effects such as respiratory depression, nausea, vomiting, itching, and inconsistent analgesic efficacy [3,4,5]. The erector spinae plane block (ESPB) was first described in 2016 for thoracic neuropathic pain [6]. The erector muscles of the spine consist of a group of three muscles (spinalis, longissimus, and iliocostalis) located on the deep side of the back. Separated at the cranial part of the back, they join to form a common mass at the sacrum level. Cadaveric studies have confirmed the blockade at the dorsal rami of multiple spinal nerves above and below the injection site when the dye is injected below the fascia of the erector spinae muscle [7,8,9]. The ventral rami are blocked inconsistently and could be involved in the analgesic effects of ESPB without extension to the paravertebral zone. The presence of the thoracolumbar fascia facilitates the local anesthetic spread in the caudal and cephalad directions. It was reported that ESPB was successfully used for spine surgery in adults [10,11,12]. However, even with ultrasound guidance, identifying bone markers as anatomical landmarks in neurogenic and congenital scoliosis patients is challenging.

We aimed to provide effective perioperative pain control and achieve intraoperative hemodynamic stability without compromising neuromonitoring with ESPB. The benefits of this approach are illustrated in six pediatric patients undergoing congenital or neurogenic scoliosis correction.

## 2. Patients and Method

Written informed consent was obtained from the parents/caregivers of the patients for this scientific contribution.

Six pediatric patients with congenital or neurogenic scoliosis underwent posterior spinal fusion involving 5 to 12 vertebral levels. The patients were American Society of Anesthesiology classes 1–3.

An hour before the surgery, dexamethasone was administered intravenously (IV) at 8 mg. In addition, all patients received 7.5 mg midazolam p.o. thirty minutes before surgery. General anesthesia with endotracheal intubation and volume-controlled ventilation (O_2_/Air 40:60) was induced and maintained using IV infusions of propofol 80–130 mcg/kg/min, remifentanil 0.05–0.1 mcg/kg/min, titrated to achieve hemodynamic stability monitored through radial artery line, and adequate anesthetic depth (BIS, GE Healthcare, Helsinki, Finland) values between 45–65. In addition, the combination of intraoperative 15 mg/kg acetaminophen, 15 mg/kg metamizole, and 10 mg/kg ibuprofen was applied as a multimodal analgesia protocol in the opioid-sparing anesthetic regimen.

**Figure 1:**
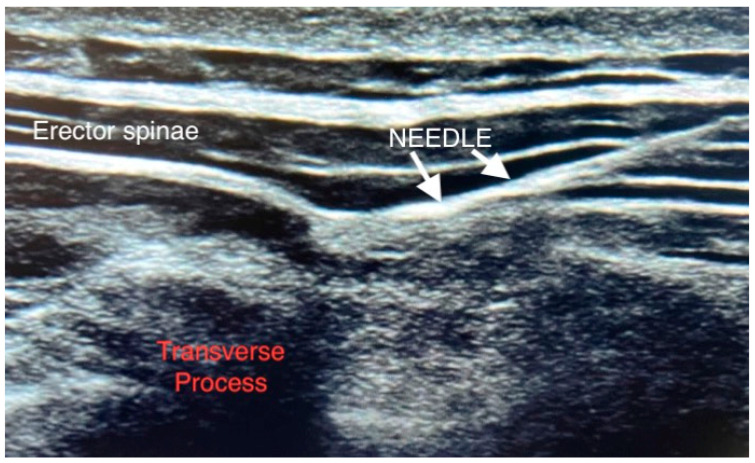
Sonoanatomy of erector spinae plane block.

After the induction of general anesthesia, bilateral, bi-level single-injection ESP blocks were performed at appropriate vertebral levels by an experienced regional anesthesiologist (Figure 1). These levels were chosen by dividing the extent of the planned incision into two and injecting at the approximate midpoint of each half. In each ESP block, a 22-gauge needle (Stimuplex Ultra 360, B Braun Melsungen AG, Germany; 80 mm) was inserted into one plane of linear array ultrasound transducer longitudinally positioned across the apex of the transverse process. The hand was directed caudally at a higher level and craniocaudally at a lower level. Penetration of the fascial plane between the transverse process and the erector spinae was confirmed using hydrolocation with 1–2 mL of 0.9% isotonic saline, followed by injection of 0.2% ropivacaine using an in-plane technique. Local anesthetic solution doses were calculated according to the patients’ weights and not to exceed a total ropivacaine dose of 3 mg/kg (Figure 1).

During the procedure, the basic hemodynamic parameters, opioid/propofol consumption, after the ESP block, the SSEP (somatosensory evoked potentials), and the time of the surgery were monitored.

Postoperative analgesia consisted of intravenous acetaminophen 15 mg/kg 6-h, 15 mg/kg metamizole 6-h, and 10 mg/kg mg ibuprofen 6-hourly administered at the same time to prevent rebound pain. In addition, the bolus of 25 μg/kg morphine sulfate and then an infusion of 10 μg/kg/h morphine sulfate was administered for rescue analgesia if the NRS score was higher than 4.

After the procedure, the basic hemodynamic parameters, opioid consumption, and NRS pain scores were monitored. During the stay at the ICU, the postoperative NRS score was observed at 0, 2, 6, 10, 14, 18, 24, and 48 h. The NRS scores were monitored daily from the second postoperative day in the pediatric orthopedic ward. In addition, possible block complications were observed.

## 3. Results

Six patients: one boy and five girls, aged 11–16 (mean 12 +/−2) years old, weight 26–68 (mean 51.7 +/−15.9) kg, height 130–160 (mean 152.9 +/−15.9) cm. The patients’ ASA class was 103, including one with no comorbidities, two obese children with mild restriction in spirometry, one with surgically treated ASD (atrial septum defect), one with Rett syndrome and asthma, and one with cerebral palsy. All patients underwent posterior spinal fusion due to congenital kyphoscoliosis in four patients and neurotic kyphoscoliosis in two patients. In addition, five patients received single-shot bilateral, bi-level ESP blocks, and one patient received single-shot bilateral, one-level ESP blocks, as seen in Table 1.

## 4. During the Surgery

The hemodynamic status of all patients was carefully monitored and stabilized throughout surgical incision, dissection, and retraction of dorsal muscles, insertion of pedicle screws, and ventricle connecting rods. After the ESP block, the SSEPs (somatosensory evoked potentials) were monitored during all surgical procedures. No SSEP change from baseline was observed. During operation, SSEP amplitude decreased by no more than 50%, and latency increased by 10%.

Opioid and propofol consumption: The total requirements of remifentanil and propofol are listed in Table 2 and vary between 0.106–0.222 (0.144 +/−0.04) μg/kg/min of remifentanil and 0.04–0.07 (0.06 +/−0.01) mg/kg/min of propofol. No patients required intraoperative intravenous remifentanil boluses. The procedure time varies between 175 and 465 (mean 242.5 +/−113.0) minutes (Table 2).

## 5. After the Surgery

The basic hemodynamic parameters were stable in the normal range. Patients were asked to rate perceived pain at all postoperative time points using the 11-point NRS (0 indicating no pain and 10 indicating the worst pain imaginable) at 0, 2, 6, 10, 14, 18, 24, 48, 72, 96, and >96 h. The evaluation was performed during the child’s examination by two independent physicians, who encouraged the patients to describe the pain intensity numerically at certain stages of observation, as shown in Table 3. The NRS score was 0 during the stay in the ICU in all cases. However, in the following days, on the pediatric orthopedic ward, the NRS score varied between 0 and 4 (1.83 +/−1.40) in the first 48 h, 0 and 3 (1.83 +/−1.40) in the third 24 h, 0 and 2 (1.25 +/−1.22) in the fourth 24 h, 0 and 2 (0.92 +/−1.00) in the fifth 24 h, and 0 and 2 (0.5 +/−0.90) in and over sixth 24 h.

## 6. Opioid Consumption

The total opioid consumption varied between 0.11 and 0.69 (0.44 +/−0.23) IV hydromorphone milligram equivalents/kg in the ICU, 0.25 and 1.11 (0.6 +/−0.34) IV hydromorphone milligram equivalents/kg in the first 24 h, 0.19 and 1.11 (0.43 +/−0.34) IV hydromorphone milligram equivalents/kg in the second 24 h, 0.08 and 0.42 (0.29 +/−0.12) IV hydromorphone milligram equivalents/kg in the third 24 h, 0 and 0.29 (0.13 +/−0.15) IV hydromorphone milligram equivalents/kg in the fourth 24 h. Patients did not require opioids on the fifth day after surgery, as in Table 4.

There were no complications related to the nerve block.

All patients were discharged home on day 5 or 6 after surgery.

## 7. Discussion

Most existing studies concern pain treatment following idiopathic scoliosis surgery. It is hard to make conclusions based only on idiopathic scoliosis treatment. Patients undergoing posterior spinal fusion for correction of idiopathic scoliosis require intravenously administered opioids for the first 36 h and report moderate-to-severe pain in the days following surgery [13,14]. Also, neurogenic and congenital scoliosis treatment is associated with severe pain [15,16,17]. This brief report suggests that ESP blockade, in combination with the intraoperative use of multiple nonopioid analgesic therapies, can be useful in pain treatment following congenital and neurogenic scoliosis surgery. We chose the ESP block for pain management due to some critical limitations of intrathecal or epidural injections. Intrathecal or epidural opioid injections and surgically inserted epidural catheters are alternative local anesthetic strategies. The duration of analgesia from intrathecal opioids is dose-dependent, limited to 12–24 h, and must be weighed against side effects such as pruritus, nausea and vomiting, sedation, and respiratory depression. Epidural opioids have similar side effects and may be less effective [18]. Epidural anesthesia by injection of local anesthetic is resource intensive, and concerns include epidural opioid side effects, hypotension, and leg weakness [19]. Pain relief is often incomplete, with significant benefits only when two catheters are placed [20]. Postoperative analgesia is probably due to the extent of surgery and surgical disruption of the epidural space. Local anesthetic wound infiltration at closure is a simple and commonly used option. However, according to a meta-analysis, the analgesic effect was modest and not evident after the first few hours after surgery [21]. On the other hand, ESPB provides adequate analgesia with fewer side effects by blocking the ventral and dorsal branches of the spinal nerves that pass through the fascial plane where local anesthetic is deposited [22,23,24]. Similar to our study, a single injection of 10 mL of local anesthetic spreads at least four to six vertebra levels at the level of the erector spinae, even in patients with major spine deformities. Furthermore, an ESP block performed before the incision minimized the need for intraoperative opioids, pain windup, and central sensitization, providing prophylactic analgesia [25]. Thus, ESPB may be a choice for preemptive analgesia.

Neurophysiological monitoring of spinal integrity is essential for the safety of scoliosis surgery.

Physical signs of paravertebral epidural diffusion of local anesthetic have been reported with ESPB [9,26], but consistent with other reports, there was no impairment of evoked potential monitoring. Therefore, we decided to perform ESP blocks mainly due to no influence on neuromonitoring. Selvi et al. [27] reported unexpected motor weakness as a side effect of the ESPB in a 29-year-old patient after a cesarean delivery operation. However, there was no motor weakness in our patients.

Neurophysiological monitoring of spinal integrity of motor evoked potential (MEP) and somatosensory evoked potentials (SSEPs) is essential for the safety of scoliosis surgery [28,29]. Anesthetics, including remifentanil and propofol, decrease the amplitude of transcranial motor evoked potentials in a dose-dependent manner [30]. Therefore, our case series demonstrates that spinal surgery’s safety should reduce anesthesia injection. However, there is a potential risk that local anesthetic can spread to the epidural or paravertebral space in ESPB [31]. We did not observe this in our case series. Our preliminary analysis shows a relationship between the intensity of the stimuli used to induce MEP and the BIS level, with recordings yielding the best results when kept at an average of 55.

Similar to other studies, hypotension associated with local anesthetic sympathectomy was not reported [32]. Therefore, the most likely explanation is that the amount of local anesthetic reaching the epidural space is insufficient to produce a clinically detectable effect. However, this should be considered if there is a high risk of intraoperative neuropathy.

We performed the ESP blocks after anesthetic induction and prone position. This slightly risks the procedure but is acceptable in young and highly anxious patients. Preoperative performance requires using a single injection block rather than a sequential technique. We chose not to place a catheter surgically at the time of wound closure to minimize the uncertainty regarding the adequacy of the craniocaudal spread in the currently disrupted tissue plane and the complexity of the postoperative analgesic regimen. Studies in other patient populations have shown that a single injection of ESP block provides effective pain relief for at least 8–12 h [33,34]. This limitation of analgesic duration can be fixed by combining intraoperative multimodal regimens with agents individually shown to reduce postoperative pain scores and opioid requirements for up to 48 h [35], which we accomplished by adding dexamethasone before the ESP block. Preemptive multimodal analgesia has significantly improved pain relief in spine surgery [36,37]. Intravenous dexamethasone prolongs the duration of the local anesthetic effect [38], and it has a systemic analgesic effect, reducing postoperative pain scores and opioid consumption for 24–48 h [39].

The main limitation of this study is the sample size and the heterogeneity of the sample size. However, due to the limited number of studies concerning pain management following neurogenic and congenital scoliosis surgery, we decided to describe ESPB as an analgesic strategy for corrective scoliosis surgery.

## 8. Conclusions

ESPB in patients undergoing congenital and neurogenic scoliosis correction surgery seems to be an essential analgesic technique that may reduce both severities of pain and opioid consumption. Further studies, including randomized controlled trials, are warranted to confirm these preliminary observations and investigate whether the strategy provides similar opioid-sparing analgesia in other types of spine surgery in pediatric patients.

## Figures and Tables

**Table 1:** Summary of clinical details. (F—female; M—male).

Description	1st	2nd	3rd	4th	5th	6th	Mean (SD)
Age (years) gender	11 Female	11 Female	12 Female	11 Female	13 Female	16 Male	12 (2)
Weight (kg)	68	55	45	68	26	48	51.7 (15.9)
Height (cm)	154	155	152	160	130	166	152.9 (12.3)
Comorbidities	Obesity; spirometry: mild restriction	Obesity; spirometry: mild restriction	ASD corrective surgery (after birth) spirometry: mild restriction	None	Rett syndrome; asthma	Cerebral Palsy	N/A
Surgical procedure	Congenital kyphoscoliosis Th4-Th10 posterior spinal fusion	Congenital kyphoscoliosis Th2-L1 posterior spinal fusion	Congenital kyphoscoliosis Th3-Th10 posterior spinal fusion	Congenital kyphoscoliosis Th1-Th6 posterior spinal fusion	Neurogenic kyphoscoliosis Th3-L2 posterior spinal fusion	Neurogenic kyphoscoliosis Th6-L4 posterior spinal fusion	N/A
Bilateral ESP block level	Th 5 and Th 8	Th 4 and Th 8	Th 4 and Th 8	Th 4	Th 6 and Th 12	Th 6 and L1	N/A
The volume of local anesthetic	4 × 10 mL 0.2% ropivacaine	4 × 10 mL 0.2% ropivacaine	4 × 10 mL 0.2% ropivacaine	2 × 10 mL 0.2% ropivacaine	4 × 5 mL 0.2% ropivacaine	4 × 10 mL 0.2% ropivacaine	N/A

**Table 2:** Duration time of the surgery and doses of TIVA.

	1st	2nd	3rd	4th	5th	6th	Mean (SD)
Intraoperative medications and analgesic adjuncts	Induction: 200 μg fentanyl + 200 mg propofol TIVA: 0.126 μg/kg/min Remifentanil + 0.05 mg/kg/min Propofol	Induction: 100 μg fentanyl + 200 mg propofol TIVA: 0.106 μg/kg/min Remifentanil + 0.07 mg/kg/min Propofol	Induction: 100 μg fentanyl + 200 mg propofol TIVA: 0.222 μg/kg/min Remifentanil + 0.07 mg/kg/min Propofol	Induction: 100 μg fentanyl + 200 mg propofol TIVA: 0.147 μg/kg/min Remifentanil + 0.06 mg/kg/min Propofol	Induction: 100 μg fentanyl + 150 mg propofol TIVA: 0.160 μg/kg/min Remifentanil + 0.06 mg/kg/min Propofol	Induction: 100 μg fentanyl + 100 mg propofol TIVA: 0.104 μg/kg/min Remifentanil + 0.04 mg/kg/min Propofol	TIVA: 0.144 (0.04) μg/kg/min Remifentanil + 0.06 (0.01) mg/kg/min Propofol
Time of the surgery	160	195	465	215	175	245	242.5 (113.00)

**Table 3:** Postoperative NRS score.

Pediatric Postoperative Care Unit (First 2 Days after Surgery)							
	1st	2nd	3rd	4th	5th	6th	Mean (SD)
0–48 h	0/0	0/0	0/0	0/0	0/0	0/0	0/0
Pediatric Orthopedic Ward (from the 2nd day after surgery)							
0–24 h	0/2	0/2	2/4	0/2	2/4	1/3	1.83 (1.40)
24–48 h	0/2	0/2	2/4	0/2	2/4	1/3	1.83 (1.40)
48–72 h	0/2	0/2	1/3	0/2	0/2	0/3	1.25 (1.22)
72–96 h	0/2	0/2	0/2	0/2	0/1	0/2	0.92 (1.00)
>96 h	0/2	0/0	0/2	0/2	0/0	0/0	0.5 (0.90)

NRS score evaluated by the first/second physician.

**Table 4:** Postoperative opioid consumption.

	1st	2nd	3rd	4th	5th	6th	Mean (SD)
Opioid consumption (IV hydromorphone milligram equivalents/kg)							
ICU	0.25	0.44	0.11	0.29	0.69	0.42	0.44 (0.23)
0–24 h	0.35	0.25	1.11	0.53	0.92	0.42	0.6 (0.34)
24–48 h	0.29	0.25	1.11	0.29	0.19	0.42	0.43 (0.34)
48–72 h	0.29	0.25	0.4	0.29	0.08	0.42	0.29 (0.12)
72–96 h	0.29	0	0.2	0.29	0	0	0.13 (0.15)
>96 h	0	0	0	0	0	0	0
Average pain scores at rest/movement (NRS 0 = no pain; 10 = worst pain)							

## Data Availability

The data presented in this study are available upon request from the corresponding author.
